# Qualitative data sharing practices in clinical trials in the UK and Ireland: towards the production of good practice guidance

**DOI:** 10.12688/hrbopenres.13667.1

**Published:** 2023-02-06

**Authors:** Megan McCarthy, Katie Gillies, Nikki Rousseau, Julia Wade, Carrol Gamble, Elaine Toomey, Karen Matvienko-Sikar, Matthew Sydes, Maura Dowling, Val Bryant, Linda Biesty, Catherine Houghton

**Affiliations:** 1 School of Nursing and Midwifery, University College Cork, Cork, Ireland; 2 Health Services Research Unit, University of Aberdeen, Aberdeen, UK; 3 Leeds Institute of Clinical Trials Research, University of Leeds, Leeds, UK; 4 Bristol Medical School, University of Bristol, Bristol, UK; 5 Health Data Science, University of Liverpool, Liverpool, UK; 6 School of Allied Health, University of Limerick, Limerick, Ireland; 7 School of Public Health, University College Cork, Cork, Ireland; 8 MRC Clinical Trials Unit at UCL, Institute of Clinical Trials and Methodology, UCL, UK; 9 BHF Data Science Centre, Health Data Research UK, London, UK; 10 School of Nursing and Midwifery, University of Galway, Galway, Ireland; 11 No particular affiliation, No particular affiliation, UK

**Keywords:** qualitative, data sharing, trials, focus groups

## Abstract

**Background**: Data sharing enables researchers to conduct novel research with previously collected datasets, thus maximising scientific findings and cost effectiveness, and reducing research waste. The value of sharing, even de-identified, quantitative data from clinical trials is well recognised with a moderated access approach recommended. While substantial challenges to sharing quantitative data remain, there are additional challenges for sharing qualitative data in trials. Incorporating the necessary information about how qualitative data will be shared into already complex trial recruitment and consent processes proves challenging. The aim of this study was to explore whether and how trial teams share qualitative data collected as part of the design, conduct, analysis, or delivery of clinical trials.

**Methods: **Phase 1 involved semi-structured, in-depth qualitative interviews and focus groups with key trial stakeholder groups including trial managers and clinical trialists (n=3), qualitative researchers in trials (n=9), members of research funding bodies (n=2) and trial participants (n=1). Data were analysed using thematic analysis. In Phase 2, we conducted a content analysis of 16 participant information leaflets (PIL) and consent forms (CF) for trials that collected qualitative data.

**Results:** Three key themes were identified from our Phase 1 findings: ‘ *Understanding and experiences of the potential benefits of sharing qualitative data from trials’, ‘Concerns about qualitative data sharing’*, and ‘ *Future guidance and funding*’. In phase 2, the PILs and CFs received revealed that the benefits of data sharing for participants were only explained in two of the study documents.

**Conclusions:** The value of sharing qualitative data was acknowledged, but there are many uncertainties as to how, when, and where to share this data. In addition, there were ethical concerns in relation to the consent process required for qualitative data sharing in trials. This study provides insight into the existing practice of qualitative data sharing in trials.

## Introduction

The demand for research data to be shared and made accessible has increased in recent years; driven by political actors, funders, and science itself ( Steinhardt *et al*., 2021). Healthcare data sharing provides evidence for better and safer care which may help strengthen care coordination, improve quality and safety, and reduce costs ( Bates *et al*., 2014; Lounsbury *et al*., 2021). In recent times, the United Kingdom (UK) has witnessed a surge in the use of secondary health care data which has generated population-based evidence to inform the delivery of better care, especially in the area of mental health ( Bates *et al*., 2014; Lounsbury *et al*., 2021). Onward data sharing facilitates researchers to conduct novel research with previously collected datasets, thus maximising scientific findings and cost effectivenesswhich should help reduce research waste ( DuBois *et al*., 2018). The benefits of sharing quantitative data in clinical trials is well established, with a controlled access approach advised ( Sydes *et al*., 2015), and guidelines exist for how this should be done ( Institute of Medicine, 2015; Keerie *et al*., 2018; Ohmann *et al*., 2017; UK CRC, 2021). While sharing quantitative data has challenges there are additional challenges for qualitative data ( NASEM, 2020) for example, making data accessible ( Steinhardt *et al*., 2021). There are certain challenges in sharing qualitative data from interviews, focus groups and observations because they tend to more readily identifiable than quantitative data. Therefore, concerns related to privacy become challenging, specifically regarding pseudonymisation which has been recognized as a major obstacle to data sharing ( Aitken *et al*., 2016; Ruggiano & Perry, 2019). Furthermore, while society may assume or anticipate their quantitative data will be shared, they may not be clear or at ease with sharing their qualitative data ( Aitken *et al*., 2016). Similarly, the public may not fully understand what qualitative data sharing is or what it entails with some qualitative researchers arguing that it is impossible to ensure that participants know what they are consenting to when it comes to qualitative data sharing ( Aitken *et al*., 2016).

Generally, adding qualitative data and analysis improves the design, conduct, analysis, and reporting of clinical trials ( Rapport *et al*., 2013). This improves the value of quantitative trials and contributes to the future development of multi-method trials ( Rapport *et al*., 2013). Qualitative elements of trials are beneficial for improving the internal and external validity of trials, assisting implementation of trial results by helping replicability of interventions in the real world and transferability of trial results to other settings, and facilitating understanding of trial findings ( O'Cathain *et al*., 2014; Rapport *et al*., 2013). In addition, qualitative components of trials can address recruitment and retention issues which are not easily addressed by quantitative methods ( Hennessy *et al*., 2018) and provide guidance to inform researchers on how to improve the process of how people are recruited trials which is a perseveringobstacleshallenge to trialists ( Hennessy *et al*., 2018; O'Cathain *et al*., 2014). While qualitative methods can be used at all stages of a trial by development or reporting, qualitative methods can be particularly helpful in developing and evaluating complex interventions as it provides valuable insights to issues experienced by potential participants ( Rapport *et al*., 2013). While qualitative research in trials has provided valuable additional understanding (optimising trial conduct and contextualising findings) relevant to individual trials, there may be missed opportunities to draw out additional insights in cross-trial analyses. Where such cross-trial analyses have been conducted, valuable findings have been generated, for example, ( Turner *et al.*, 2017), identified important differences between the experiences of intervention and control arm participants, additional to the treatment received. However, such analyses are uncommon and typically have come from several trials conducted by one group, rather than from data sharing from trials conducted by different research teams ( Rooshenas *et al*., 2016).

Despite these benefits, the archiving, provision, and reuse of data is not yet a widespread practice in qualitative research ( Hollstein & Strübing, 2018). This is mainly due to the intertwined nature of the data collection, processing, analysis, and interpretative steps of qualitative research ( Steinhardt *et al*., 2021). Moreover, there is a lack of guidance on how to approach sharing this type of data. Guidance from the UK Economic and Social Research Council applies to sharing qualitative data generally ( UKCRC, 2021). Yet, qualitative research in trials faces particular challenges in data sharing, including the need to integrate the requiredinformation and consent into recruitment processes which are already complicated ( Steinhardt *et al*., 2021; Tsai *et al*., 2016). An increasing number of researchers now face calls to “open up” qualitative research data for scientific purposes, but even if they have aninterest in doing so, they are unsure on how best to proceed ( Steinhardt *et al*., 2021). Consequently, researchers lack appropriate and clear guidance on how to conformwith data sharing guidelines in a way that provides adequate anonymity protections ( Tsai *et al*., 2016). Similarly, researchers also face the challenge of deciding whether and which of their own data can be shared and be made available for reuse, and how this should be done ( Tsai *et al*., 2016)

The aim of the “QualShare” study was to explore whether and how trial teams share qualitative data as part of the design, conduct, analysis, or delivery of trials. Hence, by exploring current qualitative data sharing practices in trials, this article will provide the foundation for further methodological work and the future production of guidance which may help improve future qualitative data sharing practices in trials. In this article, we report the key themes identified from semi-structured, in-depth qualitative interviews and focus groups with key stakeholder groups in relation to qualitative data sharing practices. The interviews and focus groups explored attitudes to data-sharing, potential benefits, and challenges of same, and participants’ recommendations regarding what guidance is needed to support those involved in sharing qualitative data. We also report the results of a content analysis of participant information leaflets (PIL) and consent forms (CF) for qualitative data collected as part of the conduct of clinical trials.

## Methods

A two-phase study was conducted to explore the existing practice of sharing of qualitative data in trials and the current issues and opportunities.

### Phase 1

***Study design.*** This study employed a qualitative descriptive approach using thematic analysis of data ( Braun & Clarke, 2006). This approach explores general beliefs and views that expose the experiences described by target populations ( Al Dandan *et al*., 2019). The perspectives and beliefs of participants were gathered using semi-structured focus group interviews via Zoom video conferencing platform.

***Recruitment.*** We aimed to recruit approximately 30 individuals who had experience of qualitative data in clinical trials either as researchers, participants in trials, or as funders reviewing grant applications for clinical trials. Participants were recruited by contacting the UK Clinical Research Collaboration (UKCRC), Irish Clinical Research Facilities (CRF) and through Health Research Board Trials-Methodology Research Network (HRB-TMRN) and MRC-NIHR Trials Methodology Research Partnership (TMRP) networks. This sample size was chosen in line with guidance from Vasileiou *et al.* (2018) regarding ‘data adequacy’, whereby we aimed to have sufficient data for meaningful analysis by capturing the perspectives of those involved in qualitative research in clinical trials. We asked the individuals who were responsible for managing the networks social media platforms to circulate a recruitment email through their respective mailing lists and social media channels.

Once prospective participants expressed an interest, they were sent the PIL and were required to complete an online CF prior to their focus group and/or interview. All participants were informed of the interview procedures and the recordings at least one week in advance of the research study. Participants were provided with contact information for the research assistant (MMC) if they had any questions in advance, and it was emphasised that consent in the research study was completely voluntary. Informed consent was obtained prior to any data collection. As the focus of our research was to determine whether and how qualitative data should be shared, we therefore did not ask for consent to share the raw data for this study.

We obtained approval for this study on the 17/02/2021 by our local Research Ethics Committee. REC number: 2021.01.009. All study materials can be found as *Extended data* ( Houghton *et al*., 2021).

***Data collection.*** This study employed online methods of data collection as face-to-face contact was not possible due to coronavirus disease 2019 (COVID-19) restrictions active at the time, and virtual focus groups offered the opportunity to bring people together who were not geographically co-located. Focus groups and interviews were conducted virtually using a secure Zoom video conferencing account and were audio-visually recorded. All interviews and focus groups were conducted by experienced qualitative researchers (CH and LB). CH is co-chair of Qualitative Research in trials Centre (QUESTS) embedded within the HRB-TMRN and is a senior lecturer in the School of Nursing and Midwifery in University of Galway. CH has extensive experience in, and has published on, qualitative methodology, qualitative evidence synthesis (QES) methodology, data collection, ethics, rigour, paradigms and analysis in qualitative research. LB is also a senior lecturer in the School of Nursing and Midwifery in University of Galway. LB is co-lead of the QES strand within ESI and her QES portfolio lies in advancing the synthesis on recruitment to trials in healthcare. Participants were made aware that focus group data could not be withdrawn once the interview was finished, but they were not obliged to answer certain questions if they were not comfortable doing so. Participants were also given the option of participating in an individual interview where participation in a focus group was not feasible. Participants did not have any relationship established with the research team members prior to study commencement. Participants were informed about the research purposes during preliminary contact, through the information leaflet and when informed consent was obtained.

Data were collected between May 2021 and July 2021. We developed a semi-structured interview guide for both the interviews and focus groups (see Supplementary File 5) that explored perspectives of sharing qualitative data collected within trials, potential benefits, and challenges of same, and recommendations for what guidance is needed to support those involved in sharing qualitative data in trials.

***Data analysis.*** We analysed the interview data using thematic analysis ( Braun & Clarke, 2006). Thematic analysis is an inductive approach to analysis, going beyond description into interpretation thus telling a coherent story about what is going on in the data ( Clarke & Braun, 2018, p106). Thematic analysis was conducted in line with the six key steps outlined by Braun and Clarke.

The research team agreed the coding and theme development from the qualitative phase to ensure the data was represented sufficiently in the developed themes which helped minimise researcher bias. The analysis was conducted by CH and MMC, supported by MD and LB and managed within QSR NVIVO version 12 to provide a transparent audit trail of the decisions made through the analysis ( Houghton *et al.*, 2013). The coding was an iterative process, and CH along with other members of the research team including MD and LB moved between the transcripts and the codes created. MD and LB also offered feedback on interpretations of the data and encouraged reflexivity. A codebook was created within QSR NVIVO to exhibit the reliability and credibility of our findings.

### Phase 2

***Study design.*** A content analysis of PILs and CFs was conducted for qualitative data collected as part of the conduct of clinical trials.

***Data collection.*** We contacted trials managers and individual researchers involved in using qualitative data in trials and the UKCRC, Irish CRF, HRB TMRN and MRC-NIHR TMRP networks to gather PILs and CFs related to qualitative components in trial. A purposive and convenience sampling method was used to collect the study documents (i.e., the PILs and CFs). A tailored data extraction form ( Table 1) was developed to extract information such as whether specific clauses for data sharing are included in the CF.

**Table 1.** Content analysis of PILs and CFs for qualitative data collected as part of the conduct of clinical trials.

Study ID	1	2	3	4	5	6	7	8	9	10	11	12	13	14	15	16
Participant Information and Consent:																
Purpose of the research	yes	yes	yes	yes	yes	yes	yes	Yes	yes	yes	yes	yes	yes	yes	yes	yes
Confidentiality of information/ how confidentiality will be protected	yes	yes	yes	yes	yes	yes	yes	Yes	yes	yes	yes	yes	yes	yes	yes	yes
Details of the consent process	yes	yes	yes	yes	yes	yes	yes	No	yes	yes	yes	yes	yes	yes	yes	yes
Data Sharing Items:																
Future publishing and reuse of the data	yes	yes	yes	yes	yes	yes	yes	Yes	yes	yes	yes	yes	yes	yes	yes?	yes?
How data will be used in publications	no	yes	yes	yes	yes	yes	no	yes	yes	yes	yes	yes	yes	no	yes	yes
Explained benefits of data sharing to participants	no	no	no	no	no	no	no	no	no	no	yes	yes	no	no	no	no
Conditions under which access to the data may be granted to others	yes	yes	yes	yes	yes	RR*	unclear	yes	no	unclear	yes	yes	no	unclear	no	no
Usage of the data during research and storage	yes	yes	yes	yes	no	yes	yes	yes	yes	no	yes	yes	yes	yes	yes	yes
How data will be de- identified in practice	yes	yes	yes	yes	yes	yes	yes	yes	yes	yes	no	yes	yes	yes	yes	yes
Indication that qualitative data could be used for further research	yes	no	no	no	yes	yes	yes	yes	yes	yes	yes	yes	yes	yes	no	no
Process for deleting data if participant wishes	no	no	yes	yes	yes	no	yes	yes	no	no	no	no	no	no	no	yes
Whether qualitative data will be deposited in a repository	no	no	no	no	no	no	no	no	no	no	yes	no	no	no	no	no
When retention of personal information expires	yes	yes	yes	yes	no	yes	no	yes	no	yes	yes	no		yes	yes	yes

***Data analysis.*** Content analysis was used to analyse whether and how consent was being obtained for qualitative data sharing ( Elo & Kyngäs, 2008). Content analysis is a valuable method for analysing qualitative material and seeks to analyse data in view of the meanings someone attributes to them ( Krippendorff, 2018). This provided baseline information on the prevalence of qualitative data sharing as well as the strategies being employed to do so. We also analysed the purpose for which the sharing of qualitative data was being requested. For this reason, we analysed the PILs and CFs for each study together for cohesion across the informed consent process.

## Results

### Phase 1

We conducted four focus groups and three individual interviews with key stakeholders (n=15): trial managers and clinical trialists (n=3), qualitative researchers in trials (n=9), members of research funding bodies (n=2) and trial participants (n=1) who have been involved in qualitative research. The majority of the participants were from the UK (n=12) and the remainder were Irish (n=3). Almost all the participants were female (n=13, male n=2). Focus groups one and two were conducted with qualitative researchers in trials (n=6). Focus group three was conducted with clinical trialists or trial managers (n=3). Focus group four was conducted with a trial participant (n=1) and members of research funding bodies (n=2). All three individual interviews were conducted with qualitative researchers (n=3). Collecting interview and focus group data arising from discussion amongst participant groups added to the variation and depth of the overall data. The interview duration ranged from 25–60 minutes. All efforts were made to ensure that all participants of the focus groups were asked the same questions as the individual interview participants, thus ensuring a pragmatic and consistent approach to data collection. Three key themes were identified from our findings. Firstly, *Understanding and experiences of the potential benefits of sharing qualitative data from trials* explores participants’ perceptions of why data sharing in trials can be useful and their experience of data sharing practices. The second theme, *Concerns about qualitative data sharing*, examines the ethical issues and potential loss of context through pseudonymisation. The third theme, *Future guidance and funding*, describes how qualitative data sharing in trials can be better supported.

***Understandings and experiences of the potential benefits of sharing qualitative data from trials.*** This theme explores participants’ general understanding of data sharing and their own experience (if any) of data sharing. For many, their views were based on knowledge of, rather than experience of, data sharing. 

Participants described their understanding of data sharing as the sharing and reusing of data outside of the research team for future research purposes:

*“I guess to me what it means is researchers collecting data and then storing it and having consent from the participants that they can then share that data with individuals outside the research team for future research”* (Focus group 1, qualitative researcher)

Many participants did not have much experience in data sharing and had very limited experience of qualitative data sharing in trials. For participants who did have experience of data sharing, this was often in the context of quantitative data sharing:

*“I know that as a unit we get lots of requests to share data from our trials and also because we do meta-analysis and IPD [Individual Participant Data] meta-analysis we often, we do a lot of asking other people to share their data but I’m not aware of the trials which have had qualitative sub-studies… So as a unit we do a lot of data sharing one way and another. But the qualitative side I have much less experience”* (Focus group 3, clinical trialist)

Participants recognised how valuable qualitative data are in the context of trials and often felt that better use of their data could be made:

*“So, I always walk away from a dataset thinking oh…there’s so much more there that I’m not making you know not valuing what they have given me enough. But in the end, you have to go where you are next going”* (Focus group 3, clinical trialist)

Many participants identified the benefits of qualitative data sharing in trials so that it can be used for other purposes, which included enabling other researchers to use it to help answer different research questions. They also recognised that data sharing for multiple purposes provides additional benefits by helping to reduce research waste for trials:

*“So, there’s limited funding for research and it doesn’t make sense for two separate groups of researchers to go out and spend the time collecting the data if one set can do that work and then another researcher can come along afterwards and don’t have to duplicate that process. So, I think reducing research waste is a big thing in trials in general so it feels like it makes sense that we should try do that with the qualitative data we are collecting as well as the quantitative data”* (Focus group 3, clinical trialist)

From a funder perspective, reducing research waste in trials had positive financial implications also:

*“Okay qualitative studies are not quite as expensive as trials, but we should be making the best we can of our resources. So, I'm certainly keen to encourage it”* (focus group 4, trial funder).

Sharing qualitative data in trials does not only have benefits for researchers but also provides opportunities to benefit research participants generally and patients in particular:

*“Thinking about the women I interviewed who were in [type of trial] and they were really keen to help research… That was really kind of important to them that’s why they took part in the trial, why they took part in the qualitative study. And so, it seems to be reflecting their wishes that we learn as much as we can from that experience to improve things for future patients”* (Focus group 3, clinical trialist)

Similarly, from the perspective of the trial participant:

*“Absolutely… I’m thinking you can lose so much just with the conclusions of a group of researchers. And also lose so much that is relevant to another set of researchers who might be looking for something else and have their antenna wanting to look for something else that wasn’t so relevant to set A, B researchers are looking for something, say in this case in a set of clinical trials”* (Interview 3, trial participant)

For some researchers, there was a sense of duty to give something back to research participants who have provided time and energy into contributing to the trial. Many participants recognised the benefits of data sharing in trials to help reduce the burden placed on participants, particularly in relation to those who may have rare diseases, which may be over researched:

*“there’s a lot of us working in particular areas and what you don’t want to do is to put more people through a process of being interviewed and researched than is needed for the question. So, if there is a way of avoiding other people having to go through it then that feels to me like another benefit … So, in the sense of not wanting to over pressurise you know often we are working with people who have health issues, so it's that sense of not wanting to over burden people”* (Focus group 3, clinical trialist).

Similarly, another aspect of qualitative data sharing is that it could potentially enhance participation to a trial by reducing patients being asked repeatedly for the same information:

“ *So, I come from a strong rare disease perspective and particularly in the rare disease populations they are so over researched. It’s exhausting for them, and then there’s a reluctance maybe to participate. So, anything that can stop that happening is very welcome”* (Focus group 4, Trial Funder)

In summary, while experience of sharing qualitative data in trials was limited, there was recognition that it could have potential benefits in terms of secondary analyses of qualitative data and making the most of available data to answer new questions and reduce research waste. However, the actual process of sharing qualitative data generally and in the context of trials did cause some concern and this is examined in the next theme.

***Concerns about sharing qualitative data from trials.*** This theme explores concerns raised by interviewees around qualitative data sharing in trials, primarily in the “how to share”, ethical considerations and the possibility of losing context through pseudonymisation.

While participants recognised the value of sharing qualitative data in trials, they did not know how or where to share their data. Participants felt that discussions around the sharing of qualitative data in trials were becoming more common, participants also highlighted the need to discuss further how to share qualitative data in trials:

*“We’ve started to think about how to share data, qualitative data within the trials. Before the emphasis has always been on the quantitative data you know lots of policies and lots of guidance around how you should manage and share quantitative data. And then thinking about qualitative data when I was trying to learn about it, I looked around and there’s things like the [funding body] and they, to my surprise, do a lot of data sharing and they have their own repository, and you can apply to use their datasets. But they weren’t into trials. I’ve not really been able to find somewhere where I could look up and access qualitative data from trials”* (Focus group 2, qualitative researcher)

Many participants expressed concerns in relation to the practical aspects of how they can generally share their qualitative data:

*“Where do we put it? I mean in [University] we’ve got a repository but within that repository it's just kind of more of a data dump than a nice, structured database which indicates all quantitative and qualitative and you know, I suppose labelling the kind of data it is and what it relates to. So, it's where we put it. It's how we let other people know that we have this data and are willing to share it”* (Focus group 2, qualitative researcher)

For some researchers, there is no system or process for identifying qualitative data that can be accessed for secondary data analysis

*“How do we as researchers know what data people have and how we can access it and what the processes are to access that because you can’t necessarily go on some university website and say, “fine I want to access your data, the qualitative data from your trials.” I would have no idea how to go about doing that”* (Focus group 2, qualitative researcher)

Participants spoke about their institutions’ data sharing approaches and/or policies. One participant spoke about the controlled access data sharing policies in their unit and highlighted the possible issues with sharing qualitative data through a repository:

*“As a unit our policy is that we have a controlled access data sharing approach. And so, people have to request the data and say why they are requesting it... the same for any of our data so the quantitative data as well as the qualitative data. And it has to be assessed by the Trial Steering Committee or the Data Access Committee to see if that request is appropriate. I think it is a different issue sticking it on a repository which anybody could look up. That’s not what we’ve got consent for, because our policy is that we do this controlled access approach. And yeah, I think that is asking a different thing”* (Focus group 3, clinical trialist).

Participants were often concerned what would happen if there were inadequate data protection controls in place which affected their choice and ability to share their qualitative data in trials:

*“My instinct about that is, that kind of open data, really open data you know where you would send the transcripts off as it were with the paper feels quite tricky for me. Immediately I don’t know what their controls are so this isn’t a reflection on the journal but that sense of, are people going shopping in the data, a little bit voyeuristic”* (Focus group 3, clinical trialist)

While, many participants highlighted the importance of data sharing, they also recognised that data sharing may need a nuanced approach which may not always be feasible:

*“I just think that kind of that you’ve got to make your data available for sharing is too black and white. And there has to be different levels of that I think, and it has to be a situation where researchers can say actually in this case it’s not appropriate because I won’t be able to get the data or people wouldn't participate or you know I mean I can’t think of examples off my head. So yeah, I just would hate it to become something that we are all expected to do, I think it’s a really great thing to do but I would like there to be clauses I guess where they accept that actually on that occasion it’s not appropriate or suitable”* (Focus group 1, qualitative researcher)

In terms of ethics, many participants expressed views on the rigorous consent process required for qualitative data sharing in trials. One trial participant explained the detailed and long-winded process involved in giving their consent for a trial and for further use of their data:

*“The consent on the first occasion permitted a second approach, the consent form had something like, one of the boxes they had to tick was that I'm happy for people to approach me again. And I think the implication was in case I had something more to say about the first study, but it didn’t say that. It said I'm happy to be approached again. And then the second study had its own consent form and it said I understand you have approached me because of the first one, I'm now consenting to a second one. So, it was a few years ago, but it was pretty rigorous”* (Focus group 4, trial participant)

Obtaining informed consent was viewed with high importance by participants. Many spoke about the importance of making sure that research data can be made available for future reuse, and as such it was essential to seek consent for future reuse of research participants’ data.

*“So yeah, getting the consent, what kind of consent we have is something that is always playing on our minds. Whether it's the broad consent that includes this as well as future research. And the implications for that. I remember when we were talking about that we were trying look up other forms of consent whether we go back each time and that didn’t seem feasible at all”* (Focus group 2, qualitative researcher)
*“I crucially would want to know that it was going to happen and to give my consent to it happening. I would want to sign on the dotted line somewhere”* (Interview 3, trial participant)

The complex and challenging consent process was also discussed in the context of how time consuming it can be when preparing to share qualitative data from trials:

*“The other challenge that I’ve come across, two of them, the amount of paperwork that is required within trials and one of the trials [name of the drug trial] so they are subject to even stricter regulations than other types of trials. So, there’s so much paperwork that has to be done and created and reviewed and approved and then changed and so on. There’s a reluctance to, if we don’t keep that to a minimum do we need that bit of extra information and do we need them to consent to that”* (Focus group 2, qualitative researcher)

It was also recognized that qualitative data sharing poses special structural challenges for ethical data practices within trials. for example, a challenge in one trial was in relation to receiving ethical approval for participants who registered and consented to participate but refused to be randomised:

*“I know in one of our trials we had huge difficulties getting through ethics for one of the qualitative elements of it, which was with people who had refused to be randomised. And you know the prospect of actually adding in layers onto our consent when getting ethics in the first place was so complex. The trial was almost easy, it was all the other bits that the ethics committee was really struggling with is I think a challenge. Because you don’t want to not be able to get through ethics but equally you want to be able to maximise what you can do”* (Focus group 3, clinical trialist)

While many participants identified concerns in relation to seeking consent and ethics, they also recognised that there have been considerable improvements in the quality and content of documents such as consent forms.

*“I have to say that our consent forms are massively improved over time, at first, I didn’t communicate that properly before. Over time I feel like we have been nearly there with how we can word it or at least it’s up to date with what it has to be. In order to satisfy journal requirements, funder requirements. It’s just us the way all of us in the same teams seem to be is that we look at that and we still worry that it isn’t you know”* (Focus group 2, qualitative researcher)

Participants also mentioned that governance has strict guidelines in relation to data sharing which they must abide by, particularly in relation to ensuring pseudonymisation (often referred to by participants as anonymisation) of research participants:

*“Whereas we'd go back to governance, and they were much more of the mindset of like we need to make sure this is completely anonymised if you are going to be sharing it outside. I suppose there were points within the consent form as well where you were talking about anonymised data and talking about anonymising transcripts. Whereas you’ve got the raw data as well”* (Focus group 1, qualitative researcher)

While the importance of pseudonymisation was understood, some participants had concerns about losing context through this process. Several participants referred to the importance of anonymising raw transcripts to remove any identifiable information. It was important that the study participant could not be identified but also certain events, trial sites or clinical settings. There was a concern that participants may not be as open and honest when being interviewed if they knew that their full transcripts would be shared:

*“Your worries might be that if people know that the entire transcript is going to be out there then that might make them behave differently”* (Interview 2, qualitative researcher)

The pseudonymisation of qualitative data would be easier for large multiples site trials, but in smaller or single site trials or trials with a particular context, for example rare diseases, it may be more challenging to pseudonymise. Many participants discussed the importance of context in qualitative research; and that important context could be lost through pseudonymisation:

*“You know the whole point of qualitative is that its meaning in context. Do we want to lose the context of what the researcher brings to it?... I think if the transcripts are there it could even in some cases be quite hard you know how much of it would you need to cut out. And if I'm coming to that with fresh eyes, would it even make sense”* (Interview 1, qualitative researcher)

For one researcher who had previously shared qualitative data from trials, it was important to provide some context to make the analysis meaningful. Using a controlled access approach to sharing would enable this:

“ *So, if it was controlled as [name] spoke about and you know where it's going, and you have written guarantees of confidentiality from where it's going to etc. You can maybe open that context up a little bit to offer, for example with participants gender, participants age band, which country they are in”* (Focus Group 3, clinical trialist).

Similarly, broader sharing outside of agreed teams was considered potentially difficult due to lack of context, and this was of particular issue when conducting qualitative research in large complex trials:

*“And that was quite tricky because it was about developing complex interventions which are evaluated in trials. But actually, we had anonymised it so much because not just the person but the intervention and the treatment that I think you lose some of the grittiness which was quite hard”* (Focus Group 1, qualitative researcher)

It was acknowledged by some participants that qualitative researchers can be quite protective of the primary participants, and data that has been generated through developing a unique relationship between researcher and participant.

*“I think as qualitative researchers we are often quite protective of our datasets; we often feel very privileged with the accounts that we get access to. And then it kind of feels I think quite uncomfortable the idea of publishing anonymised transcripts alongside my paper”* (Focus group 1, qualitative researcher).
*“Depending on who’s publishing the research there might be some aspect of protecting the participant”* (Interview 2, qualitative researcher).

In summary, many of the concerns identified centred around uncertainty and need for guidance around qualitative data sharing generally and in the context of trials. The need for guidance is explored further in the next theme.

***Future guidance and funding for sharing qualitative data from trials.*** This theme explores the issues around guidance and funding for qualitative data sharing purposes in trials. Across the focus groups, many participants expressed concerns over the lack of guidance and funding available for qualitative data sharing aspects in trials.

Many participants shared their worries about an absence of clear standards and established guidelines explaining where, when and how to share qualitative data from trials. This issue was at the forefront of many participants experiences:

*“I think there’s a lot that funders can do to encourage focus on these issues. Not quite sure what form that might take but somewhere there should be a statement of good practice” (focus group 6, trial funder)*

An urgent need for clear guidance and good practice when pseudonymising qualitative data from trials was highlighted:

*“But yeah, I feel what you need is a little checklist when you are going through your transcripts to anonymise them here are things which could go towards identifying your participants”* (Focus group 3, clinical trialist)

For some, guidance in the format of clear and practical examples of supporting documents such as CFs and PILs would be extremely helpful and valuable to enable the process of qualitative data sharing from trials. Sample consent forms were identified as a practical resource to help guide researchers in the right direction:

*“I think the wording that goes into the consent form you know what words people have used in the past and just simple good examples that people have”* (Focus group 4, trial funder)

While many participants expressed a great need for clear guidance for trials, others felt that there may already be guidance in place that specifically addresses qualitative data sharing in the context of trials. However, they did not know where to look or who to turn to for guidance:

*“I mean it may be that it's already there and I just didn’t know where to look. I didn’t know that I needed to look if you see what I mean. So, there may be something already, but you know that would be another place for things to go”* (Focus group 2, qualitative researcher)

Moreover, many participants identified the importance and necessity of planning for data sharing from the beginning of the trial design. Participants recognised that planning for qualitative data sharing is not always easy and data sharing can often be a complex process:

*“And thinking about it well in advance which is something all of us struggle to do. It's at the time of publication that we start thinking about it, I think that’s a huge challenge”* (Focus group 2, qualitative researcher)

In addition to the need for clear and practical guidance, participants expressed a need for the availability of sufficient funds for the sharing of qualitative data. This was particularly in the context of pseudonymising data such as interview transcripts which is time consuming and has resource implications for the funding of data sharing. For some, the sharing of qualitative data would not be possible without sufficient funds and resources:

*“I think we need the infrastructure to anonymise the data, funds around that, I think we have to start costing for the funds. But also, storage facilities, repositories, data management guidance about how we index it and make this available and file all the data so other people can come in and use it and use it appropriately. So, it’s like we need that infrastructure and funding as well as just being willing to share the data because without that its, you can’t really do it to be honest”* (Focus group 1, qualitative researcher)

Participants also recognised the additional time involved in the thorough pseudonymisation of qualitative data and some identified that this needs to be considered in advance of applying for and writing grant applications.

*“Building funding into grants to be able to prepare data for sharing, so you know about the different software and the time it takes to prepare data. I think at the moment it done for quantitative there’s money built in but not necessarily for the qualitative and I think that might be an area that needs to be looked at. Does it require additional resources?”* (Focus group 2, qualitative researcher)

In summary, participants called for easily accessible guidance and resources for how best to share qualitative data in trials. They also felt the cost and time of data sharing process need to be acknowledged through funding.

### Phase II

Overall, 16 PILs and 16 CFs were received from clinical trial assistants, qualitative researchers in trials, clinical trialists and trial managers. Feasibility trials, treatment trials, screening trials and prevention trials were among the types of clinical trials conducted. The PILs indicated that the trials were broadly conducted to: evaluate treatment interventions for diseases and conditions (i.e., multiple sclerosis); explore supportive care interventions and/or programmes for chronic illnesses (i.e., cancer) and to assess ways of preventing diseases and conditions (i.e., stress and anxiety). 11 PILs and CFs were from the UK and five PILs and CFs were from Ireland. All the Irish trials were funded by the Health Research Board (HRB) and the UK trials were funded by the National Institute for Health Research (NIHR) and the Medical Research Council (MRC). Whether the documents indicated the purpose of the research, the consent process and how confidentiality will be protected was recorded and is shown in Table 1. The benefits of data sharing for participants were only explained in two of the study documents i.e., the benefits of data sharing in making research more accessible to patients. All documents described the plans for future publishing. Similarly, 13 of the 16 documents indicated how data will be used in future publications. For example, one PIL stated: “ *When all the interviews have finished, we will write up the information and publish it in scientific journals.”* (Study 15).

In total, 8 of the study documents specified the conditions under which access to the data may be granted to others directly related to the research project. For example, one consent form stated: “ *I give permission for the interview to be audio recorded and for the transcriber and delegated members of the research team to have access to the audio recording, or transcription of it, and understand that my confidentiality will be maintained”* (Study 1).

Of the 16 study documents, 15 indicated how data will be de-identified in practice. For example, one PIL stated: “ *We will then replace your name with a code and store your name separately from your other information (data). Only the researcher will hold the key to the code. The researcher will enter the information that you provide on a password-protected computer using your code*” (Study 2). Six of the study documents also described the process for deleting data if a participant wished and 12 of the study documents outlined when the retention of personal information expired.

Information regarding the qualitative data being available in a repository was only specified in one study document. Overall, 11 of the study documents indicated whether qualitative data could be used for future research. For example, one CF outlined: *“I understand that the information collected about me may be used to support other research in the future and may be shared anonymously with other researchers”* (Study 1).

## Discussion

This study sheds light into the existing practice of sharing of qualitative data in trials and the current issues and opportunities. In phase one, participants identified a range of benefits and concerns associated with qualitative data sharing in trials, while also identifying many recommendations for improving how we go about sharing qualitative data in trials. In phase two, a content analysis of PILs and CFs found that data sharing items generally tend not to be specified, for example, the benefits of data sharing for participants were only explained in two of the study documents.

In phase one, participants identified numerous benefits associated with qualitative data sharing in trials. In line with previous research ( DuBois *et al*., 2018; Mozersky *et al*., 2020), these benefits included the potential to increase transparency of research and enable secondary users to explore new research questions or collate findings across multiple studies. As such, qualitative data sharing helps reduce research waste by maximizing the value of data that is often expensive and resource intensive to collect. Furthermore, by enabling researchers to use existing data rather than collect new data, participants recognised the benefits of qualitative data sharing for trial participants by reducing the trial participant burden. This mirrors previous work ( Rapport *et al*., 2013) which found that many participants supported and consented to qualitative data sharing, with helping others identified as the primary motivation for agreeing to share their data.

Despite the many advantages of sharing qualitative research data in trials, the practice appears to be relatively uncommon. Participants across the focus groups and interviews had very limited experience of both data sharing generally and data sharing in trials with views often being based on knowledge of, rather than experience of data sharing. This is similar to findings from one United States (US) study which found that onward sharing of qualitative data generally remains rare, with only 4% of qualitative researchers in the US having ever shared qualitative data into a repository ( Mozersky *et al*., 2021). While many participants expressed their support for qualitative data sharing in trials, many do not have experience of sharing their qualitative research data, possibly due to the challenges reported by participants.

Qualitative data sharing is associated with many ethical challenges ranging from anonymity, informed consent and confidentiality which are well documented in the literature ( Alexander *et al*., 2020; Bishop, 2009). For pseudonymisation, participants discussed the extensive resources and time required to remove all identifying information from their data when preparing to share qualitative data in trials. This reflects previous research that highlighted the time-consuming nature of pseudonymisation data and transferring data to file formats that best preserve it and that are accepted by a repository ( Cliggett, 2013; Saunders *et al*., 2015). In line with previous research ( Aitken *et al*., 2016; Campbell *et al*., 2007; Chalmers & Muir, 2003), participants also highlighted data sharing concerns in trials which related to the potential loss of context through pseudonymization. Anonymizing can have a limiting impact for certain trials by potentially removing spatially explicit and rich information ( Campbell *et al*., 2007). Therefore, it can be difficult to completely anonymise data while still leaving it in an analysable form.

Informed consent processes, which require researchers being clear to research participants how the information gathered will be used, stored, and shared, may also prevent researchers from sharing qualitative data within trials. Participants key concerns related to obtaining informed consent, ensuring participants agreed with the study procedures, and not breaching trust. Participants highlighted data sharing concerns in relation to the consent process which they described as ‘ *complex,’* ‘ *lengthy’* and ‘ *difficult.’* Obtaining informed consent from participants for sharing their data with others and then reusing it for purposes other than those for which it was originally intended for was also identified as a concern by participants. As highlighted in the literature, participants generally tend to volunteer to share their experiences for one specific research question ( Chauvette *et al.*, 2019; Thorne, 1998). Hence, reusing their data for a different research question may infringe on the conditions under which consent was obtained in the first place unless further consent is received for additional analyses ( Thorne, 1998). Therefore, participants highlighted the importance of researchers ensuring clear and transparent informed consent that communicates data sharing plans at the outset of a study. This could significantly help facilitate qualitative data sharing and be acceptable to participants. Furthermore, even when consent is granted and the sharing and re-use of human-participant data is possible, further ethical concerns may arise in the re-use process ( Alexander *et al*., 2020). Participants spoke about concerns of a lack of engagement among original research participants in subsequent research and highlighted that it cannot be assumed that research participants would want their data to be re-used.

While participants highlighted challenges and concerns concerning qualitative data sharing, they were still supportive of it and identified many recommendations for improving how we go about sharing qualitative data. Participants indicated that they would be more willing to share their data if there was clear guidance on sharing qualitative data, for example the mechanisms by which to share data such as repositories; if they received sufficient funds for the additional work involved; and if they received clear guidance on ethics, consent-related issues and anonymisation in trials. Firstly, participants key concerns were related to the lack of guidance and information available for researchers to share qualitative data in trials. Similarly, one study carried out ( Rodriguez *et al*., 2022) found that there are no standardised set of recommendations on how to anonymise clinical trial datasets for qualitative data sharing. A significant observation identified from this study was the participants’ desire for more information about data sharing in relation to ‘ *how can we share,’* ‘ *what can we share’* and ‘ *where can we share.’* Many of the participants felt that researchers lack knowledge of the current research practices and the governance systems and structures in place. There also remains a lack of standards for metadata and documentation to facilitate qualitative data re-use, and many data repositories that support open access do not have adequate standards to ensure the appropriate and accurate re-use of qualitative data in future research ( Antes *et al*., 2018; Mozersky *et al*., 2021).

In addition to the need for clear and practical guidance, participants expressed a need for the availability of sufficient funds for the sharing of qualitative data which would enhance their willingness to share qualitative data. This was particularly in the context of pseudonymising qualitative data such as interview transcripts. Presently, researchers must look through their data and remove any potential identifiers which is both time consuming and labour intensive. In addition, as outlined by Mozersky *et al*. 2021, there are no standards specific to qualitative data to determine when it is adequately de-identified. Finally, as outlined previously, participants discussed the lack of clear guidance in relation to the consent process. Participants, therefore, expressed the need for researchers to receive template consent forms. Going forward, it is important for qualitative researchers to ensure clear and transparent informed consent that communicates data sharing plans at the outset of a study as this could significantly facilitate qualitative data sharing in trials and be acceptable to participants.

### Strengths and limitations

To the best of our knowledge, this study provides the first account of views and experiences of clinical trialists, trial managers, qualitative researchers, funders, and trial participants about sharing qualitative data in trials. While a few other studies have explored views and experiences of qualitative data sharing among researchers ( Mozersky *et al*., 2020), we believe ours is the first to explore views and experiences of qualitative data sharing in the context of trials among trialists, trial managers, qualitative researchers, funders, and trial participants. The findings of our study have implications for qualitative researchers more broadly given the international shift towards sharing qualitative data, which has historically not been shared. A key limitation of our study was the sample size which may affect the transferability of our findings. We aimed to recruit approximately 32 participants, however, only 15 participants were recruited across the focus groups and interviews. It is also recognised that most research participants in this study were qualitative researchers (n=9). Further research is therefore needed with members of research funding bodies, trial managers, clinical trialists and participants who have participated in a clinical trial. Moreover, we only interviewed one trial participant, which may be attributed to our online recruitment strategy due to COVID-19 restrictions. Due to COVID-19 restrictions, in person-centred recruitment strategies appeared challenging, primarily due to data protection concerns. Thus, we opted for a virtual recruitment approach which may have not been as inclusive and engaging for trial participants who had been involved in a qualitative study. However, few studies have found that conducting qualitative research virtually can be just as effective as in person ( Keen *et al.*, 2022). In addition, we recognise that the sample size of sixteen study documents (PILs and CFs) is small. However, these documents were only intended to give an indication of the current consent procedures for qualitative data collected as part of the conduct of clinical trials. In addition, the informed consent procedures for the included studies were not linked with study participants so we were unable to conduct any comparative analyses across Phases 1 and 2. Finally, the purposive and convenience sampling method used to collect the documents in this study may have led to an enthusiasm bias. Therefore, investigators interested in qualitative data sharing, and better alignment with good practice, were probably more likely to contribute documents.

## Conclusion

In recent years, data sharing in research is becoming increasingly normative and the demand for research data to be shared and made available and accessible has become louder ( Steinhardt *et al*., 2021). The key reasons that speak in favour of data sharing include the participants personal benefit, research benefit and reduction of research waste and participant burden. However, despite these reasons and the growing movement toward providing open access to data precipitated by requirements of some funding bodies, it may not always be appropriate to share qualitative data as concerns and risks also exist. The main concerns related to data sharing include the sensitive nature of data, time and cost implications, complex consent processes, ethical issues, and the potential loss of context through pseudonymization. It is important that researchers make informed decisions about which data should be open for sharing and consider implications of this on future research. Furthermore, there is a substantial need for easily accessible guidance and resources for how best to share qualitative data in trials. Additional methodological research on how best to consent for sharing qualitative data and how to effectively share qualitative data without losing the importance of context is needed.

## Data Availability

The purpose of our study was to explore whether and how qualitative data is shared within trials. Before the completion of the study, we felt it would not be appropriate to share raw data collected for this study and therefore did not obtain consent to do so. We used NVIVO coding queries function as advocated by Tsai *et al*. (2016) to facilitate transparency and the credibility of our analysis ( Houghton *et al.*, 2013) (See Table 2). The data cannot be shared via an alternative route of closed access, as we did not gain consent for others, beyond the research team, to access the data. Readers who wish to ask questions about the data can contact Catherine Houghton who can provide a visual overview of the analysis as opposed to sharing the raw transcripts. We can also offer partial access to the PILs and CFs analysed in this study on request. Open Science Framework: Qualitative data sharing practices in clinical trials in the UK and Ireland: Towards the production of good practice guidance. https://doi.org/10.17605/OSF.IO/BKC8D ( Houghton *et al*., 2021). This study contains the following extended data: Appendix 1: Recruitment emails Appendix 2: Participant Information leaflets Appendix 3: Informed consent form Appendix 4: Distress Protocol Appendix 5: Focus group Interview guide Data are available under the terms of the https://creativecommons.org/licenses/by/4.0/legalcode. (CC-BY 4.0)
