# Supplementary material for: Qualitative data sharing practices in clinical trials in the UK and Ireland: towards the production of good practice guidance
Source: HRB Open Res. 2023 Feb 6;6:10. [Version 1] doi: 10.12688/hrbopenres.13667.1 (PMC10345597; doi:10.12688/hrbopenres.13667.1)
Supplement: Peer review history

# Peer review: Reviewer response for version 1

**DOI:** 10.21956/hrbopenres.14946.r34166

**Published:** 2023-07-13

**Authors:** Anisa Rowhani-Farid

**Affiliations:** 1 Independent Researcher, Baltimore, Maryland, USA

This is an important study as it explores the importance of sharing qualitative data in trials. The researchers interviewed a variety of participants including funders, trial managers and trialists, researchers and trial participants. The sample size is low but they have acknowledged this limitation. My only recommendation is that the authors contact the interviewees and ask for their permission to share their data as it would be good practice for a qualitative study on data sharing to share its qualitative data. Since the sample size is only 9, this should be achievable. If the participants disagree, then this can be shared in the paper. The paper reads well however it was somewhat lengthy and the discussion provided a nice summary of the findings but a bit repetitive of the results section at times. I would consider shortening the results section.

Is the work clearly and accurately presented and does it cite the current literature?

Yes

If applicable, is the statistical analysis and its interpretation appropriate?

Not applicable

Are all the source data underlying the results available to ensure full reproducibility?

No

Is the study design appropriate and is the work technically sound?

Yes

Are the conclusions drawn adequately supported by the results?

Yes

Are sufficient details of methods and analysis provided to allow replication by others?

Yes

Reviewer Expertise:

data sharing, clinical trial reporting, meta-research, open science

I confirm that I have read this submission and believe that I have an appropriate level of expertise to confirm that it is of an acceptable scientific standard.

# Peer review: Reviewer response for version 1

**DOI:** 10.21956/hrbopenres.14946.r33472

**Published:** 2023-03-06

**Authors:** Nicola Howe

**Affiliations:** 1 Newcastle Clinical Trials Unit, Newcastle University, Newcastle upon Tyne, UK

This paper provides a good succinct introduction to the rational for including qualitative data collection in trials and also to data sharing and sharing of qualitative data in particular. Data analysis and collection is well described with enough detail or reference to the papers detailing the approach taken so that I believe this study would be replicable. There are some interesting comments presented from the focus groups and interviews categorised into what appear to be appropriate themes. The results of the Content Analyses are surprising and help to demonstrate the authors points that there is a very real need for a strategy on best practice or guidance on sharing of qualitative data - I wonder if this could be replicated in a larger study? I agree that one limitation of this study is that only one “research participant” was interviewed. I have no comment on the availability of the underlying data; I agree with the authors it would not be appropriate to share this. Overall, I find that this paper is very clearly written and subsequently I have very few outstanding questions or comments. However, I have several points that the authors may wish to address:

- Typo “perseveringobstacleshallenge“ in second para of introduction.
- Very minor observation but should “qualshare” study be referred to first in the abstract or title before the last para of the introduction to make it clear that this paper is from the qualshare study?
- Results - phase 1: Consider a re-structure of the first sentence - I had to read this a few times to understand. Also, was there any cross over between the 15 stakeholders interviewed and those included in the focus groups - I think not but would the authors be able to clarify/make clear in this paragraph?
- In the discussion, use of “participants” to mean those who took part in this study can feel a little confusing as if talking about research participants although I see that the authors make the distinction in para 5 using “original research participants”. Perhaps consider “stakeholders” or “the research participants”?

Is the work clearly and accurately presented and does it cite the current literature?

Yes

If applicable, is the statistical analysis and its interpretation appropriate?

Not applicable

Are all the source data underlying the results available to ensure full reproducibility?

Partly

Is the study design appropriate and is the work technically sound?

Yes

Are the conclusions drawn adequately supported by the results?

Yes

Are sufficient details of methods and analysis provided to allow replication by others?

Yes

Reviewer Expertise:

data sharing, clinical trials, participants' attitudes towards data sharing, anonymisation, data management

I confirm that I have read this submission and believe that I have an appropriate level of expertise to confirm that it is of an acceptable scientific standard.
